# Supplementary material for: Diagnostic accuracy of the WHO clinical definitions for dengue and implications for surveillance: A systematic review and meta-analysis
Source: PLoS Negl Trop Dis. 2021 Apr 26;15(4):e0009359. doi: 10.1371/journal.pntd.0009359 (PMC8102005; doi:10.1371/journal.pntd.0009359)
Supplement: S1 File — (DOCX) [file pntd.0009359.s001.docx]

**Diagnostic accuracy of the WHO clinical definitions for dengue and implications for surveillance: a systematic review and meta-analysis**

**Methodology**

PRISMA checklist for this systematic review is provided in S7 Table.

**Assessing risk of bias:**

Risk of bias was assessed using a modified version of the QUADAS-2 tool (S2 Table) looking at the following domains: patient selection, index test, reference standard, and patient flow. Two independent reviewers (NR and SL) answered each question with yes, no, or unclear; any disagreements were resolved by a third reviewer (RM). A domain was deemed at high or unclear risk of bias if the answer to any question was no or unclear, respectively. A study was deemed at high risk of bias if any domain was at high risk.

**Data extraction:**

For all eligible records, the following information was extracted: study design, time period, location(s), inclusion/exclusion criteria, clinical definition assessed, reference standard(s), total number of patients, number in final analysis (with reasons for exclusion). 2x2 tables of diagnostic accuracy (i.e. true positive, false positive, true negative, false negative) were used as the principal summary measures. Where studies assessed both definitions, separate 2x2 tables were constructed. Data was extracted by one reviewer and verified by a second reviewer (NR and SL). Any disagreements were resolved by a third reviewer (RM). Authors were contacted for missing information, and if no response was received within 3 weeks this was repeated. If no response was subsequently received, it was recorded as not specified.
