# Supplementary material for: Diagnostic accuracy of the WHO clinical definitions for dengue and implications for surveillance: A systematic review and meta-analysis
Source: PLoS Negl Trop Dis. 2021 Apr 26;15(4):e0009359. doi: 10.1371/journal.pntd.0009359 (PMC8102005; doi:10.1371/journal.pntd.0009359)
Supplement: S1 Table — (DOCX) [file pntd.0009359.s002.docx]

**S1 Table: Search strings used in systematic review.**

| **Database** | **Search string** |
| --- | --- |
| **PubMed** | (sensitivity and specificity[MeSH Terms] OR evaluat* OR sensitiv* OR specific* OR utility OR accura* OR "Diagnosis, Differential"[MeSH Terms] OR "Predictive Value of Tests"[MeSH Terms]) AND ("Guidelines as Topic"[MeSH Terms] OR "Government Agencies/standards"[MAJR] OR classif* OR definition* OR guideline* OR guidance) AND (who OR world health organisation OR world health organization OR world health organization[MeSH Terms]) AND ("Dengue/diagnosis"[MAJR] OR dengue OR denv OR DHF OR DSS OR dengue shock syndrome OR dengue haemorrhagic fever) |
| **EMBASE** | (evaluat*.mp. or evaluation/ or diagnostic test accuracy study/ or "sensitivity and specificity"/ or sensitiv*.mp. or specific*.mp.or utility.mp. or diagnosis/ or differential diagnosis/ or diagnostic accuracy/ or accura*.mp.) and (guideline*.mp.or guidance.mp.or classification/ or classif*.mp.or definition*.mp.) and (world health organisation.mp. or world health organization.mp. or world health organization/ or WHO.mp.) and (dengue shock syndrome/ or dengue.mp. or dengue hemorrhagic fever/ or dengue/ or Dengue virus/ or DHF.mp. or DSS.mp.) |
| **Scopus (MEDLINE/ EMBASE records excluded)** | TITLE-ABS-KEY ( ( evaluat*  OR  sensitiv*  OR  specific*  OR  utility  OR  diagnos* )  AND  ( classif*  OR  definition*  OR  guideline*  OR  guidance )  AND  ( who  OR  "world health organisation"  OR  "world health organization" )  AND  ( dengue  OR  denv  OR  dhf  OR  dss  OR  "dengue shock syndrome"  OR  "dengue haemorrhagic fever" ) )  AND  PUBYEAR  >  1996  AND NOT  INDEX ( medline  OR  embase  ) |
| **OpenGrey** | (evaluat* OR sensitiv* OR specific* OR utility OR diagnos*) AND (classif* OR definition* OR guideline* OR guidance) AND (who OR “world health organisation” OR “world health organization”) AND (dengue OR denv OR DHF OR DSS OR “dengue shock syndrome” OR “dengue haemorrhagic fever”) |
