# Supplementary material for: Diagnostic accuracy of the WHO clinical definitions for dengue and implications for surveillance: A systematic review and meta-analysis
Source: PLoS Negl Trop Dis. 2021 Apr 26;15(4):e0009359. doi: 10.1371/journal.pntd.0009359 (PMC8102005; doi:10.1371/journal.pntd.0009359)
Supplement: S2 Table — (DOCX) [file pntd.0009359.s003.docx]

**S2 Table:** **Modified QUADAS-2 quality assessment tool.**

| **Domain** | **Specific questions** |
| --- | --- |
| **Patient selection** | Was a consecutive or random sample of patients enrolled? |
|  | Did the study avoid inappropriate exclusions? |
| **Index test** | Were patients classified according to WHO criteria without knowledge of the results of the reference standard? |
| **Reference standard** | Is the reference standard likely to classify the target condition correctly? |
|  | Were the reference standard results interpreted without knowledge of clinical diagnosis? |
| **Patient flow** | Is the time period between reference standard and clinical diagnosis short enough to be reasonably sure that the dengue status did not change between the two? |
|  | Were uninterpretable/intermediate test results reported? |
|  | Were withdrawals from the study explained? |
