# Supplementary material for: Diagnostic accuracy of the WHO clinical definitions for dengue and implications for surveillance: A systematic review and meta-analysis
Source: PLoS Negl Trop Dis. 2021 Apr 26;15(4):e0009359. doi: 10.1371/journal.pntd.0009359 (PMC8102005; doi:10.1371/journal.pntd.0009359)
Supplement: S3 Table — (DOCX) [file pntd.0009359.s004.docx]

**S3 Table: Anticipated effect of study bias on sensitivity/specificity estimates.**

| **Study** | **Effect on Sn** | **Effect on Sp** | **Rationale** |  |
| --- | --- | --- | --- | --- |
| Sawasdivorn 2001[1] | ?SEN- | ?SPEC+ | Unclear if ELISA reference standard was unpaired – if so could overestimate specificity and underestimate sensitivity through more reference standard false positives |  |
| Martinez 2005[2] | 0 | 0 | Low risk of bias |  |
| Gan 2011[3] | ? | ? | Conference abstract so difficult to assess risk of bias |  |
| Lagi 2011[4] | ? | SPEC+ | Unpaired IgM ELISA reference standard could overestimate specificity, but study was carried out in a non-endemic population which may offset this.  Conference abstract therefore difficult to assess risk of bias. |  |
| Fonseca 2012[5] | SEN+ | SPEC+ | Unpaired IgM ELISA would overestimate specificity.  Convenience sample may lead to more ‘classical’ cases being included which would overestimate sensitivity |  |
| Nujum 2012[6] | SEN- | SPEC+ | Small number of dengue-positive cases could underestimate sensitivity (missing one case would have a large effect).  Unpaired IgM ELISA reference standard may overestimate specificity and underestimate sensitivity. |  |
| Capeding 2013[7] | SEN- | SPEC+ | Unpaired IgM ELISA reference standard may overestimate specificity and underestimate sensitivity |  |
| Daumas 2013[8] | SEN- | SPEC+ | Unpaired IgM ELISA reference standard may overestimate specificity and underestimate sensitivity |  |
| Gutiérrez 2013 [14] – cohort study | 0 | 0 | Low risk of bias |  |
| Gutiérrez 2013[9] – Hospital study | SEN+ | SPEC+ | Patients were receiving frequent regular blood tests, including dengue tests. If clinicians aware of blood results would improve accuracy of clinical diagnosis. |  |
| Gan 2014[10] | ? | ? | Unclear risk of bias |  |
| Nujum 2014[11] | SEN- | SPEC+ | Unpaired IgM ELISA reference standard may overestimate specificity and underestimate sensitivity |  |
| Pitisuttithum 2015[12] | SEN- | ? | Case-control recruitment may underestimate sensitivity by picking out non-classical cases.  Unreliable serology (not from acute episode) may lead to both false negatives and false positives. |  |
| Nealon 2016[13] | 0 | 0 | Low risk of bias |  |
| Seshan 2017[14] | SEN- | SPEC+ | Unpaired IgM ELISA reference standard may overestimate specificity and underestimate sensitivity. |  |
| Caicedo 2019[15] – Aedes Network | 0 | 0 | Low risk of bias |  |
| Caicedo 2019[15] – National Public Health Surveillance | 0 | 0 | Low risk of bias |  |
| **MODIFIED CRITERIA** | | | | |
| Peragallo 2003[16] | SEN- | SPEC- | Relied on retrospective recollection of symptoms by participants – may be inaccurate and would reduce sensitivity and/or specificity.  Unpaired serology not in acute illness – likely to underestimate clinical definition performance |  |
| Juárez 2005[17] | SEN- | SPEC+ | Unpaired IgM ELISA reference standard may overestimate specificity and underestimate sensitivity. |  |
| Low 2011[18] | 0 | 0 | Low risk of bias |  |
| Wieten 2012[19] | SEN+ | SPEC+ | Unpaired IgM ELISA reference standard may overestimate specificity and underestimate sensitivity. Study carried out in returning traveller population (i.e. non-endemic) so may offset this.  Only patients who received dengue serology were tested. |  |
| Ridde 2016[20] | SEN- | SPEC+ | Reduced number of symptoms in definition may reduce sensitivity.  Low levels of PCR testing.  Clinician may have been aware of RDT result. |  |
| Bodinayake 2018[21] | 0 | 0 | Low risk of bias. |  |

**Note:** Sn, sensitivity; Sp, specificity. ? indicates that the effect of the risk of bias on sensitivity/specificity is unclear; 0 indicates that the (low) risk of bias is anticipated to have no effect; SEN and SPEC refer to the effects on sensitivity and specificity, respectively, with a + suffix indicating an anticipated *over*estimation due to the risk of bias, and vice versa for – suffix.

**References**

1. Sawasdivorn, S., et al., *Efficacy of clinical diagnosis of dengue fever in paediatric age groups as determined by WHO case definition 1997 in Thailand.* Dengue Bulletin, 2001. **25**: p. 56-64.

2. Martínez, R.A., F.A. Díaz, and L.A. Villar, *Evaluation of the World Health Organization clinical definition of dengue.* Biomedica : revista del Instituto Nacional de Salud, 2005. **25**(3): p. 412-416.

3. Gan, V.C., et al., *RAPID DIAGNOSIS OF DENGUE IN A HOSPITAL-BASED COHORT.* The American Journal of Tropical Medicine and Hygiene, 2011. **85**(6_Suppl): p. 398.

4. Lagi, F., et al., *Dengue virus infection in Tuscany, Italy: Evaluation of ICT rapid test and clinical criteria for the diagnosis of acute dengue fever.* Tropical Medicine and International Health, 2011. **16**(SUPPL. 1): p. 248.

5. Fonseca, B.A., et al., *Evaluation of the performance of clinical and laboratorial dengue diagnosis during an epidemic in a medium-sized city in southeast brazil.* American Journal of Tropical Medicine and Hygiene, 2012. **87**(5 SUPPL. 1): p. 334.

6. Nujum, Z.T., et al., *Performance of WHO probable case definition of dengue in Kerala, India, and its implications for surveillance and referral.* Dengue Bulletin, 2012. **36**: p. 94-104.

7. Capeding, M.R., et al., *Dengue and other common causes of acute febrile illness in Asia: an active surveillance study in children.* PLoS Negl Trop Dis, 2013. **7**(7): p. e2331.

8. Daumas, R.P., et al., *Clinical and laboratory features that discriminate dengue from other febrile illnesses: a diagnostic accuracy study in Rio de Janeiro, Brazil.* BMC infectious diseases, 2013. **13**: p. 77-77.

9. Gutiérrez, G., et al., *Evaluation of the diagnostic utility of the traditional and revised WHO dengue case definitions.* PLoS neglected tropical diseases, 2013. **7**(8): p. e2385-e2385.

10. Gan, V.C., et al., *Diagnosing dengue at the point-of-care: utility of a rapid combined diagnostic kit in Singapore.* PLoS One, 2014. **9**(3): p. e90037.

11. Nujum, Z.T., et al., *Comparative performance of the probable case definitions of dengue by WHO (2009) and the WHO-SEAR expert group (2011).* Pathogens and global health, 2014. **108**(2): p. 103-110.

12. Pitisuttithum, P., et al., *Accuracy of Clinical Diagnosis of Dengue Episodes in the RV144 HIV Vaccine Efficacy Trial in Thailand.* PLoS One, 2015. **10**(5): p. e0127998.

13. Nealon, J., et al., *Symptomatic Dengue Disease in Five Southeast Asian Countries: Epidemiological Evidence from a Dengue Vaccine Trial.* PLoS Negl Trop Dis, 2016. **10**(8): p. e0004918.

14. Seshan, V., et al., *Serological, molecular and clinical correlates of dengue from a tertiary care centre in Chennai, India.* Archives of virology, 2017. **162**(10): p. 2983-2988.

15. Caicedo, D.M., et al., *Development of clinical algorithms for the diagnosis of dengue in Colombia.* Biomedica, 2019. **39**(1): p. 170-185.

16. Peragallo, M.S., et al., *Probable dengue virus infection among Italian troops, East Timor, 1999-2000.* Emerg Infect Dis, 2003. **9**(7): p. 876-80.

17. Juárez, J., et al., *Evaluación de la definición de caso probable de dengue clásico durante el brote de dengue en Lima, 2005.* Revista Peruana de Medicina Experimental y Salud Pública, 2005. **22**(3): p. 205-211.

18. Low, J.G.H., et al., *The early clinical features of dengue in adults: challenges for early clinical diagnosis.* PLoS neglected tropical diseases, 2011. **5**(5): p. e1191-e1191.

19. Wieten, R.W., et al., *Dengue in travellers: Applicability of the 1975-1997 and the 2009 WHO classification system of dengue fever.* Tropical Medicine and International Health, 2012. **17**(8): p. 1023-1030.

20. Ridde, V., et al., *Presence of three dengue serotypes in Ouagadougou (Burkina Faso): research and public health implications.* Infectious diseases of poverty, 2016. **5**: p. 23-23.

21. Bodinayake, C.K., et al., *Evaluation of the WHO 2009 classification for diagnosis of acute dengue in a large cohort of adults and children in Sri Lanka during a dengue-1 epidemic.* PLoS neglected tropical diseases, 2018. **12**(2): p. e0006258-e0006258.
