# Supplementary material for: Diagnostic accuracy of the WHO clinical definitions for dengue and implications for surveillance: A systematic review and meta-analysis
Source: PLoS Negl Trop Dis. 2021 Apr 26;15(4):e0009359. doi: 10.1371/journal.pntd.0009359 (PMC8102005; doi:10.1371/journal.pntd.0009359)
Supplement: S4 Table — (DOCX) [file pntd.0009359.s005.docx]

**S4 Table:** **Data from studies looking at WHO 1997 definition.**

| **STUDY** | **TP** | **FP** | **TN** | **FN** | **TOTAL** |
| --- | --- | --- | --- | --- | --- |
| Sawasdivorn 2001 | 138 | 31 | 7 | 0 | 176 |
| Martinez 2005 | 100 | 88 | 1 | 1 | 190 |
| Gan 2011 | 140 | 16 | 4 | 2 | 162 |
| Capeding 2013 | 12 | 22 | 260 | 80 | 374 |
| Daumas 2013 | 68 | 70 | 3 | 1 | 142 |
| Gutiérrez 2013 – hospital study | 425 | 1668 | 1263 | 51 | 3407 |
| Gutiérrez 2013 – cohort study | 699 | 341 | 96 | 24 | 1160 |
| Gan 2014 | 141 | 40 | 10 | 6 | 197 |
| Nealon 2016 | 92 | 16 | 2764 | 227 | 3099 |
| Caicedo 2019 – AN | 624 | 307 | 18 | 38 | 987 |
| Caicedo 2019 – PHS | 59 | 265 | 117 | 20 | 461 |

**Note:** TP, true positive; FP, false positive; TN, true negative; FN, false negative; AN, Aedes Network Study; PHS, Public Health Surveillance Network Study.
