# Supplementary material for: Diagnostic accuracy of the WHO clinical definitions for dengue and implications for surveillance: A systematic review and meta-analysis
Source: PLoS Negl Trop Dis. 2021 Apr 26;15(4):e0009359. doi: 10.1371/journal.pntd.0009359 (PMC8102005; doi:10.1371/journal.pntd.0009359)
Supplement: S5 Table — (DOCX) [file pntd.0009359.s006.docx]

**S5 Table:** **Data from studies looking at WHO 2009 definition**

| **Study** | **TP** | **FP** | **TN** | **FN** | **TOTAL** |
| --- | --- | --- | --- | --- | --- |
| Gan 2011 | 139 | 16 | 4 | 3 | 162 |
| Lagi 2011 | 29 | 27 | 46 | 7 | 109 |
| Fonseca 2012 | 800 | 347 | 174 | 169 | 1490 |
| Nujum 2012 | 5 | 171 | 76 | 2 | 254 |
| Gutiérrez 2013 – cohort study | 412 | 1314 | 1617 | 64 | 3407 |
| Gutiérrez 2013 – hospital study | 718 | 400 | 37 | 5 | 1160 |
| Gan 2014 | 142 | 37 | 13 | 5 | 197 |
| Nujum 2014 | 133 | 389 | 288 | 41 | 851 |
| Pitisuttithum 2015 | 100 | 21 | 61 | 11 | 193 |
| Seshan 2017 | 113 | 13 | 22 | 2 | 150 |
| Caicedo 2019 – AN | 636 | 315 | 10 | 26 | 987 |
| Caicedo 2019 – PHS | 64 | 314 | 68 | 15 | 461 |

**Note:** TP, true positive; FP, false positive; TN, true negative; FN, false negative; AN, Aedes Network Study; PHS, Public Health Surveillance Network Study.
