# Supplementary material for: Diagnostic accuracy of the WHO clinical definitions for dengue and implications for surveillance: A systematic review and meta-analysis
Source: PLoS Negl Trop Dis. 2021 Apr 26;15(4):e0009359. doi: 10.1371/journal.pntd.0009359 (PMC8102005; doi:10.1371/journal.pntd.0009359)
Supplement: S6 Table — (DOCX) [file pntd.0009359.s007.docx]

**S6 Table:** **Data from studies using modified WHO criteria.**

| **Study** | **TP** | **FP** | **TN** | **FN** | **TOTAL** |
| --- | --- | --- | --- | --- | --- |
| Wieten 2012 – traditional | 111 | 157 | 120 | 21 | 409 |
| Wieten 2012 – revised | 107 | 149 | 25 | 128 | 409 |
| Bodinayake 2018 | 295 | 158 | 292 | 93 | 838 |
| Peragallo 2003 | 16 | 14 | 49 | 6 | 85 |
| Juárez 2005 | 137 | 105 | 40 | 33 | 315 |
| Low 2011 – traditional | 232 | 1275 | 604 | 18 | 2129 |
| Low 2011 – revised | 237 | 1451 | 428 | 13 | 2129 |
| Ridde 2016 | 41 | 104 | 234 | 0 | 379 |

**Note:** TP, true positive; FP, false positive; TN, true negative; FN, false negative.
