# Supplementary material for: Diagnostic accuracy of the WHO clinical definitions for dengue and implications for surveillance: A systematic review and meta-analysis
Source: PLoS Negl Trop Dis. 2021 Apr 26;15(4):e0009359. doi: 10.1371/journal.pntd.0009359 (PMC8102005; doi:10.1371/journal.pntd.0009359)
Supplement: S7 Table — (DOCX) [file pntd.0009359.s008.docx]

**S7 Table:** **PRISMA checklist for systematic review.**

| **Section/topic** | **#** | **Checklist item** | **Reported on page #** | **Text excerpt** |
| --- | --- | --- | --- | --- |
| **TITLE** | | | |  |
| Title | 1 | Identify the report as a systematic review, meta-analysis, or both. | 1 | Diagnostic accuracy of the WHO clinical definitions for dengue and implications for surveillance: a systematic review and meta-analysis |
| **ABSTRACT** | | | |  |
| Structured summary | 2 | Provide a structured summary including, as applicable: background; objectives; data sources; study eligibility criteria, participants, and interventions; study appraisal and synthesis methods; results; limitations; conclusions and implications of key findings; systematic review registration number. | 2-3 | Abstract on page 2 |
| **INTRODUCTION** | | | |  |
| Rationale | 3 | Describe the rationale for the review in the context of what is already known. | 5-6 | The development of the WHO case classification has been reviewed elsewhere[13], and whilst methodologically robust, the aim was to improve early prediction of severe disease, rather than distinguish dengue from non-dengue febrile illnesses. Thus, most studies have focused on the guidelines’ prognostic value. In this systematic review, we assess the diagnostic performance of the 1997 and 2009 WHO clinical definitions of ‘probable dengue’ in febrile patients and discuss the implications for surveillance and control. |
| Objectives | 4 | Provide an explicit statement of questions being addressed with reference to participants, interventions, comparisons, outcomes, and study design (PICOS). | Table 1, p. 6 | Population: Febrile patients in dengue endemic areas  Intervention: Strict use of WHO clinical definitions of dengue (1997 or 2009)  Comparison: Confirmatory laboratory tests for dengue  Outcome: Sensitivity, specificity, and likelihood ratios of WHO clinical definitions of dengue  Study design: Systematic review |
| **METHODS** | | | |  |
| Protocol and registration | 5 | Indicate if a review protocol exists, if and where it can be accessed (e.g., Web address), and, if available, provide registration information including registration number. | 6 | The protocol was registered on PROSPERO on 27/01/2020 (CRD42020165998). The PICOS statement is outlined in Table 1. |
| Eligibility criteria | 6 | Specify study characteristics (e.g., PICOS, length of follow-up) and report characteristics (e.g., years considered, language, publication status) used as criteria for eligibility, giving rationale. | 7 | Studies comparing the WHO diagnostic criteria to a suitable reference standard (see below) in patients with unexplained fever were included.  The index tests were the 1997[11] and 2009[12] WHO clinical definitions for dengue.  With no accepted reference standard for dengue, any of the following, as per WHO guidance[12], were acceptable: IgM or IgG serology, plaque reduction neutralisation test or hemagglutination inhibition, NS1 antigen/antibody test, (RT-)PCR, or virus isolation. |
| Information sources | 7 | Describe all information sources (e.g., databases with dates of coverage, contact with study authors to identify additional studies) in the search and date last searched. | 8 | PubMed, EMBASE, Scopus, and OpenGrey were searched using the strings outlined in S1 Table. Records published from 1997 to the last search on 19/1/2020 were included, with no restrictions on type of publication or language.  Abstracts of all articles and short notes in the annual Dengue Bulletin (published by WHO SEARO) from 1997-2014 (last available volume) were also included.  Authors of conference abstracts were contacted to identify related peer-reviewed publications. Finally, all articles citing (from The Web of Knowledge) and cited by (from reference lists) included studies were screened. For articles not available on Web of Knowledge, Google Scholar was used. |
| Search | 8 | Present full electronic search strategy for at least one database, including any limits used, such that it could be repeated. | S1 Table | PubMed: (sensitivity and specificity[MeSH Terms] OR evaluat* OR sensitiv* OR specific* OR utility OR accura* OR "Diagnosis, Differential"[MeSH Terms] OR "Predictive Value of Tests"[MeSH Terms]) AND ("Guidelines as Topic"[MeSH Terms] OR "Government Agencies/standards"[MAJR] OR classif* OR definition* OR guideline* OR guidance) AND (who OR world health organisation OR world health organization OR world health organization[MeSH Terms]) AND ("Dengue/diagnosis"[MAJR] OR dengue OR denv OR DHF OR DSS OR dengue shock syndrome OR dengue haemorrhagic fever) |
| Study selection | 9 | State the process for selecting studies (i.e., screening, eligibility, included in systematic review, and, if applicable, included in the meta-analysis). | 8-9 | Titles and abstracts were independently screened by two reviewers (NR and SL). This was repeated for eligible full-text articles, with the reason for exclusion recorded. Any disagreements were resolved by a third reviewer (RJM).  Only studies using unmodified WHO criteria were included in the meta-analysis. |
| Data collection process | 10 | Describe method of data extraction from reports (e.g., piloted forms, independently, in duplicate) and any processes for obtaining and confirming data from investigators. | 8-9, S1 File | Study information and 2x2 table data were extracted by one reviewer and verified by a second reviewer (NR and SL). Any disagreements were resolved by a third reviewer (RJM).  Authors were contacted for missing information, and if no response was received within 3 weeks this was repeated. If no response was subsequently received, it was recorded as not specified. |
| Data items | 11 | List and define all variables for which data were sought (e.g., PICOS, funding sources) and any assumptions and simplifications made. | S1 File | For all eligible records, the following information was extracted: study design, time period, location(s), inclusion/exclusion criteria, clinical definition assessed, reference standard(s), total number of patients, number in final analysis (with reasons for exclusion), and 2x2 tables of diagnostic accuracy (i.e. true positive, false positive, true negative, false negative). Where studies assessed both definitions, separate 2x2 tables were constructed. |
| Risk of bias in individual studies | 12 | Describe methods used for assessing risk of bias of individual studies (including specification of whether this was done at the study or outcome level), and how this information is to be used in any data synthesis. | 8  S1 File, S2 Table | Risk of bias was assessed using a modified version of the QUADAS-2 tool (S2 Table) looking at the following domains: patient selection, index test, reference standard, and patient flow. Two independent reviewers (NR and SL) answered each question with yes, no, or unclear; any disagreements were resolved by a third reviewer (RM). A domain was deemed at high or unclear risk of bias if the answer to any question was no or unclear, respectively. A study was deemed at high risk of bias if any domain was at high risk. |
| Summary measures | 13 | State the principal summary measures (e.g., risk ratio, difference in means). | 8 | Study information and 2x2 table data (principal summary measure) were extracted |
| Synthesis of results | 14 | Describe the methods of handling data and combining results of studies, if done, including measures of consistency (e.g., I^2^) for each meta-analysis. | 9 | Meta-analysis for sensitivity, specificity, and likelihood ratios for both definitions was done using the MIDAS statistical package[15] on Stata/IC 14 (College Station, TX, USA).  Heterogeneity was assessed using the I2 and Chi-square statistics. |
| Risk of bias across studies | 15 | Specify any assessment of risk of bias that may affect the cumulative evidence (e.g., publication bias, selective reporting within studies). | 9 | Deeks’ funnel plot asymmetry test was used to detect publication bias for both meta-analyses. |
| Additional analyses | 16 | Describe methods of additional analyses (e.g., sensitivity or subgroup analyses, meta-regression), if done, indicating which were pre-specified. | N/A |  |
| **RESULTS** | | | |  |
| Study selection | 17 | Give numbers of studies screened, assessed for eligibility, and included in the review, with reasons for exclusions at each stage, ideally with a flow diagram. | 9-10, Fig. 1 | The original search identified 1471 records.  After duplicates were removed, 1088 records remained. Dengue Bulletin provided 340 additional records; the 2005 and 2006 volumes could not be found online and were not screened.  119 full-text articles were assessed for eligibility, of which 16 were included. Citation analysis identified 5 additional records. |
| Study characteristics | 18 | For each study, present characteristics for which data were extracted (e.g., study size, PICOS, follow-up period) and provide the citations. | Tables 3 and 4; pp. 11-21 |  |
| Risk of bias within studies | 19 | Present data on risk of bias of each study and, if available, any outcome level assessment (see item 12). | p.22, Fig. 2 |  |
| Results of individual studies | 20 | For all outcomes considered (benefits or harms), present, for each study: (a) simple summary data for each intervention group (b) effect estimates and confidence intervals, ideally with a forest plot. | Fig. 3-4, Table 5; pp. 22-24 | The findings for the 1997 definition are summarised in Fig 3 and S4 Table. Overall sensitivity was 93% (95% CI: 77-98, range: 13-100), and the specificity was 29% (95% CI: 8-65, range: 1-99). Positive and negative likelihood ratios were 1.3 (95% CI: 0.9-1.9) and 0.24 (95% CI: 0.12-0.50) respectively.  The findings for the 2009 definition are summarised in Fig 4 and S5 Table. The overall sensitivity was 93% (95% CI: 86-96, range: 71-99), and the specificity was 31% (95% CI: 18-48, range: 3-74). Positive and negative likelihood ratios were 1.3 (95% CI: 1.1-1.7) and 0.24 (95% CI: 0.13-0.45) respectively. |
| Synthesis of results | 21 | Present results of each meta-analysis done, including confidence intervals and measures of consistency. | pp. 22-23, Fig. 3-4 | As above |
| Risk of bias across studies | 22 | Present results of any assessment of risk of bias across studies (see Item 15). | S1 and S2 Figs, p. 22 | There was no evidence of publication bias (S1 and S2 Figs). |
| Additional analysis | 23 | Give results of additional analyses, if done (e.g., sensitivity or subgroup analyses, meta-regression [see Item 16]). | N/A |  |
| **DISCUSSION** | | | |  |
| Summary of evidence | 24 | Summarize the main findings including the strength of evidence for each main outcome; consider their relevance to key groups (e.g., healthcare providers, users, and policy makers). | pp. 25-26 | In this review, we have pooled evidence from multiple regions assessing the accuracy of the 1997 and 2009 WHO clinical definitions for diagnosing dengue fever. We have shown that both definitions have high sensitivity (93%) but poor specificity (29% and 31%). No modification improved accuracy. This makes the definitions useful rule-out criteria but unreliable as the basis for diagnosis, which is concerning given they are often used as such[8-10]. |
| Limitations | 25 | Discuss limitations at study and outcome level (e.g., risk of bias), and at review-level (e.g., incomplete retrieval of identified research, reporting bias). | 32 | The main limitation was the significant heterogeneity (in methods and results) of included studies and the high risk of bias. This is likely due to the use of different reference standards between studies. As diagnostic accuracy varies between and within confirmatory tests[6], and no test is perfect, this would introduce significant bias to results (especially when IgM or IgG serology alone were used for confirmation). |
| Conclusions | 26 | Provide a general interpretation of the results in the context of other evidence, and implications for future research. | 33 | This review has demonstrated the poor diagnostic accuracy of the clinical definitions for dengue in the absence of confirmatory testing. This has real-world costs both for treating clinicians and for surveillance systems, magnified by COVID-19. As fragile healthcare systems prepare to cope with the possibility of double epidemics, further research into improved clinical guidance, access to diagnostic testing, and accurate quantification of dengue burden and transmission will be essential. |
| **FUNDING** | | | |  |
| Funding | 27 | Describe sources of funding for the systematic review and other support (e.g., supply of data); role of funders for the systematic review. | N/A | Provided on submission and not in Acknowledgements, as per PLOS NTD guidelines |

Page numbers refer to original submitted manuscript. Adapted from: Moher D, Liberati A, Tetzlaff J, Altman DG, The PRISMA Group (2009). Preferred Reporting Items for Systematic Reviews and Meta-Analyses: The PRISMA Statement. PLoS Med 6(7): e1000097. doi:10.1371/journal.pmed1000097. For more information, visit: [www.prisma-statement.org](http://www.prisma-statement.org).
