# Supplementary figures and images for: Diagnostic accuracy of the WHO clinical definitions for dengue and implications for surveillance: A systematic review and meta-analysis
Source: PLoS Negl Trop Dis. 2021 Apr 26;15(4):e0009359. doi: 10.1371/journal.pntd.0009359 (PMC8102005; doi:10.1371/journal.pntd.0009359)

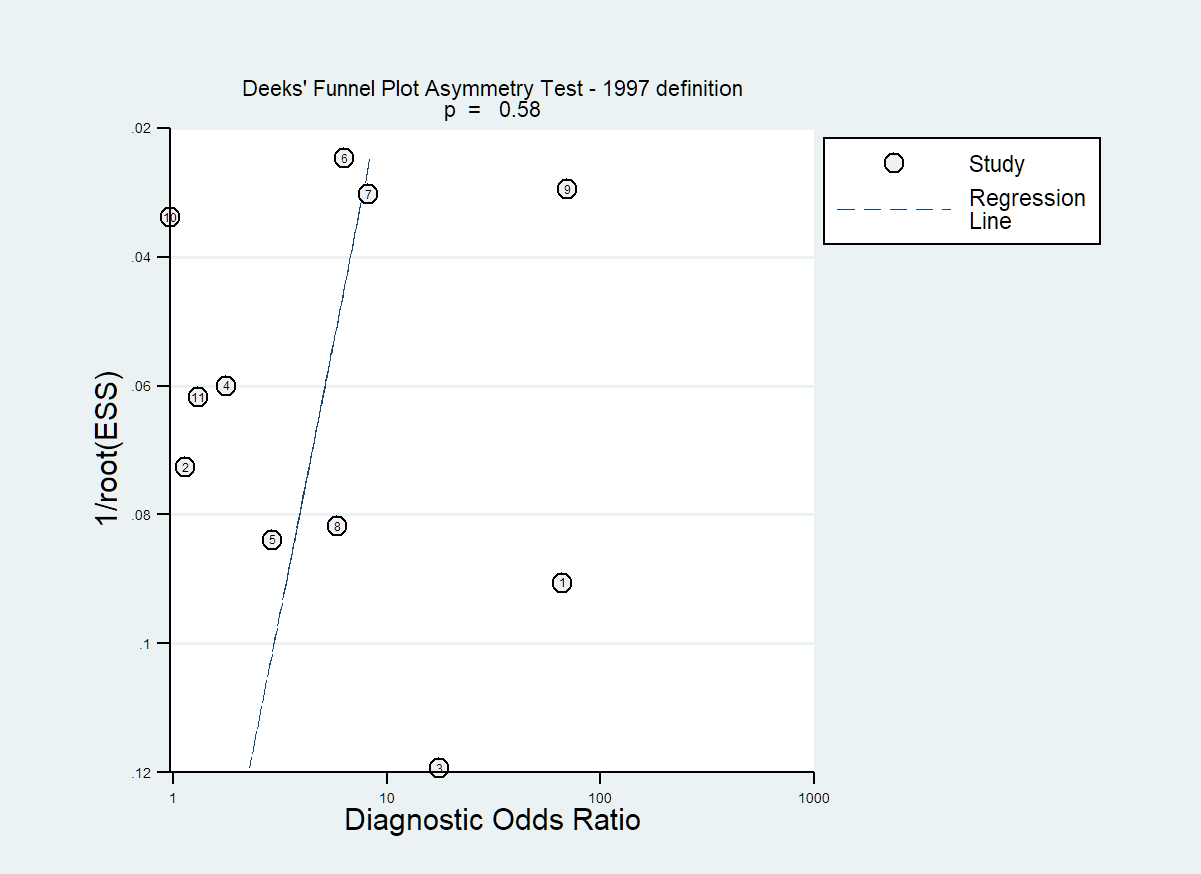

Supplement: S1 Fig — 1, Sawasdivorn 2001 [18]; 2, Martinez 2005 [19]; 3, Gan 2011 [20]; 4, Capeding 2013 [24]; 5, Daumas 2013 [25]; 6, Gutiérrez 2013 –cohort study [16]; 7, Gutiérrez 2013 –hospital study [16]; 8, Gan 2014 [26]; 9, Nealon 2016 [29]; 10, Caicedo 2019 –Aedes Network Study [17]; 11, Caicedo 2019 –Public Health Surveillance Network study [17]. (TIF) [file pntd.0009359.s009.tif]

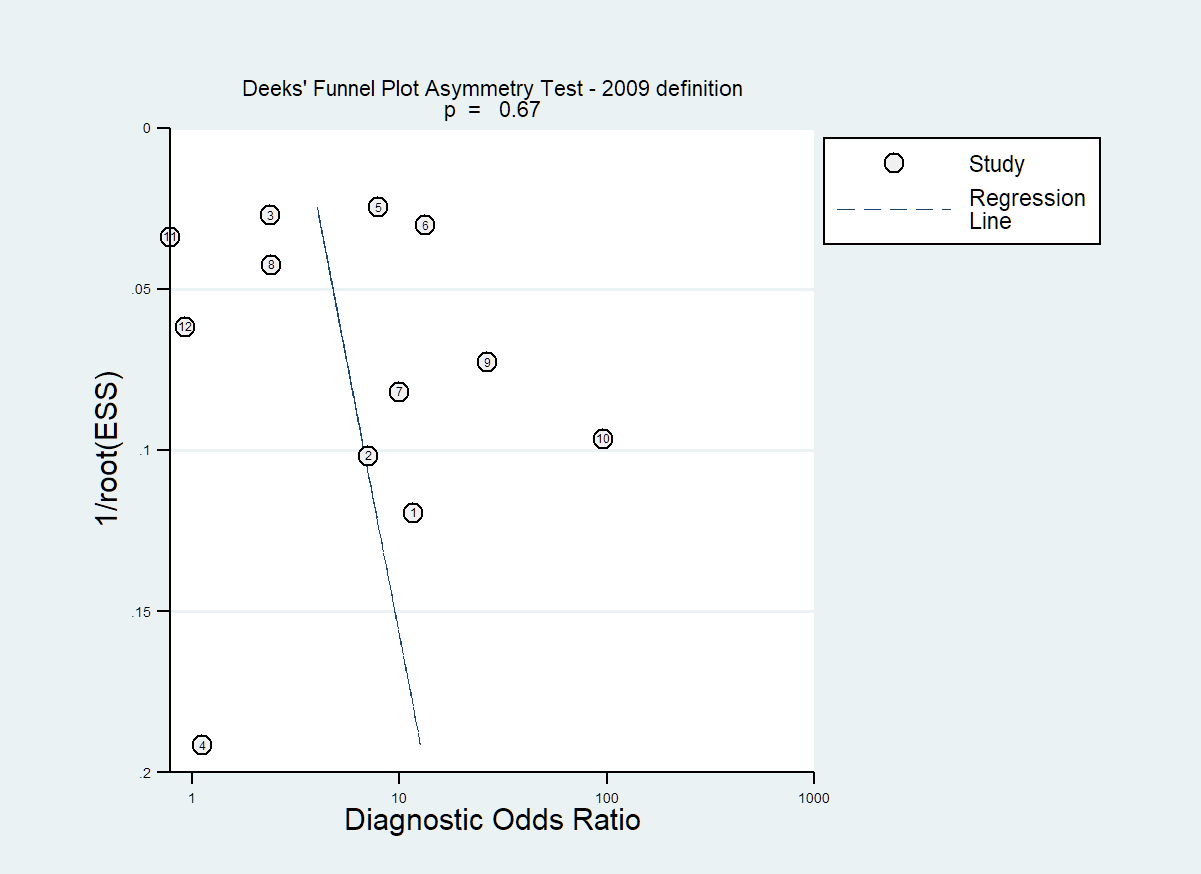

Supplement: S2 Fig — 1, Gan 2011 [20]; 2, Lagi 2011 [21]; 3, Fonseca 2012 [22]; 4, Nujum 2012 [23]; 5, Gutiérrez 2013 –cohort study [16]; 6, Gutiérrez 2013 –hospital study [16]; 7, Gan 2014 [26]; 8, Nujum 2014 [27]; 9, Pitisuttithum 2015 [28]; 10, Seshan 2017 [30]; 11, Caicedo 2019 –Aedes Network Study [17]; 12, Caicedo 2019 –Public Health Surveillance Network study [17]. (TIF) [file pntd.0009359.s010.tif]
