# Supplementary material for: Diagnostic accuracy of the WHO clinical definitions for dengue and implications for surveillance: A systematic review and meta-analysis
Source: PLoS Negl Trop Dis. 2021 Apr 26;15(4):e0009359. doi: 10.1371/journal.pntd.0009359 (PMC8102005; doi:10.1371/journal.pntd.0009359)
Supplement: Attachment — Submitted filename: PLOSNTD Response to reviewer comments_FINAL tc.docx [file pntd.0009359.s011.docx]

Dear PLOS NTDs Editorial Board,

We would like to thank you for considering our manuscript "Diagnostic accuracy of the WHO clinical definitions for dengue and implications for surveillance: a systematic review and meta-analysis" for publication. We would also like to thank the reviewers for their helpful and constructive comments. Below, we outline how we have addressed these comments in our revised manuscript, and once again welcome further comments on how it may be refined. Please note that all line numbers refer to the manuscript with tracked changes in ‘All Markup’ view.

**Methods:**

***“I am curious if [the authors] have identified studies in which the case classification performed better than expected and why this happened.”***

Two extra paragraphs have now been added to address this, lines 291-312. As overall accuracy would vary depending on dengue prevalence within study populations, we chose to address those studies that showed a higher-than-expected specificity, the Achilles’ heel of the case definitions.

***“In some studies the inclusion criteria or the age categories are not clear. I wonder how the authors handled that.”***

This has now been moved from S1 file to lines 143-145. Unfortunately, most corresponding authors could not be reached or did not respond, hence most missing information was labelled as ‘not specified’ in Tables 3 and 4.

***“It would be helpful for the authors to indicate the anticipated impact of the suspected bias…In a separate supplemental table, the authors should explain the rationale for their choices.”***

This is now outlined in a new Table S3. Unfortunately for many studies (especially conference abstracts), it was difficult to assess the risk of bias due to lack of detail in the manuscript, and in some studies the risk of bias could plausibly have both a positive and negative effect on sensitivity/specificity estimates. Where applicable, this is outlined in the rationale column.

**Conclusions:**

***“I think the authors should acknowledge that [the effect of epidemiology on clinical decision making] also plays an important role in the global reports of DENV cases and not only the clinical case classification.”***

We now address this in our limitations section in lines 429-431.

***“The authors write a lot about how the low specificity of the case classification could be harmful in the COVID-19 era. In my opinion [the authors] should be cautious with this statements, because the studies included date back from before this.”***

We have now added this caveat to our discussion of the impact of COVID-19 in lines 353-359.

***“The value of the study would be further enhanced if the authors could use their data to suggest further refinements to the classification of dengue symptoms to increase specificity.”***

Thank you for this suggestion. Although this was not the goal of our study, we did look at it briefly but found there to be insufficient data to make any firm recommendations. We discussed this on lines 380 to 406 and suggested some possible refinements but concluded that further research would be needed to do this. As we only considered studies that had used WHO criteria, to examine this fully would require a separate search to add studies which did not use these criteria. We are considering a separate systematic review on this topic.

***“The authors should discuss [the different time windows of laboratory diagnostic tests] and the extent to which the literature can address the problem.”***

We agree this is an important point and have added some text in the discussion on line 399-407.

***“A broader concern deserving discussion is reconciling the high expansion factors (e.g. 7-fold) which implies a sensitivity for the health care system of only about 14%. This discrepancy implies that the populations used for this meta-analysis are not very representative of dengue cases overall. Prospective fever studies in a given location would seem especially useful for public health purposes. The authors may wish to discuss which, if any, of the studies had that design and whether and how the results differed from the overall studies that they reviewed.”***

We would like to thank the reviewer for this very constructive and insightful comment. We did not exclude prospective fever studies provided they fit the inclusion criteria but only three out of the 23 included studies had this design. When considering this comment, we examined our included studies further and found this to be the design for the two studies which had outlying results for outliers, namely high sensitivity and low specificity, which we had not noted previously. This may be a possible explanation for them being outliers, although the third study with this design found the opposite. We have added more detailed discussion on this on lines 291-306.

***“The authors may wish to discuss the outliers, such as the study by Capeding from Indonesia.”***

As above, this is now discussed in lines 291-306. Both outlier studies were carried out in the same centres and share multiple authors, and thus confounding may exist, but they were also prospective fever studies.

**Editorial and Data Presentation:**

***“From the text it is not really clear why [distinguishing DENV-infection from COVID-19] is important, but I can imagine it is especially for the isolation measurements.”***

Apologies that this was not clear. We have revised the text accordingly on lines 352-364.

***“I am wondering in areas where testing on DENV is not common how the testing procedures on COVID-19 are.”***

This is an interesting point. Sorry that we could not find any clear studies in the literature about this. We would be very happy to add to the discussion if the reviewer has a paper or papers they could recommend. As more data on COVID-19 testing are published from dengue endemic countries, this may be something that could be looked at with a future study.

***“Line 67: ‘not unlike the ongoing COVID-19 pandemic’: I don’t understand the meaning of this line. Asymptomatic spread also contributes to the COVID-19 pandemic.”***

We have now amended this so that it reads more clearly by removing the double negative (line 70).

***“Line 73: ‘is’ should be ‘are’”***

We have now amended this (line 76).

***“304: I think with using laboratory markers it is not always difficult to distinguish bacterial infection from viral infection. Moreover, exposure plays an important role as well.”***

We have now removed that sentence from our discussion.

***“One limitation of this study is that report from the African region are missing, although I think there aren’t a lot.”***

Only one eligible study could be found from the African continent (Ridde, 2016). As mentioned, this is likely to be due to lack of suitable studies being conducted in Africa where rates of dengue remain low despite climate suitability, and as such dengue may not be a research priority compared to illnesses such as malaria. These low rates may themselves be a result of underrecognition/underdiagnosis, a point we mention in lines 429-431.

***“I would suggest that the authors replace the statement in lines 59-60 ‘as we discuss in this article.’ It would be helpful to summarize their recommendation in a phase, such as testing the borderline clinical diagnoses.”***

We have now incorporated this in lines 60-63.
